# An efficient method for the site-specific ^99m^Tc labeling of nanobody

**DOI:** 10.52601/bpr.2021.210012

**Published:** 2021-08-31

**Authors:** Qi Luo, Hannan Gao, Jiyun Shi, Fan Wang

**Affiliations:** 1 Bioland Laboratory (Guangzhou Regenerative Medicine and Health Guangdong Laboratory), Guangzhou 510005, China; 2 Medical Isotopes Research Center and Department of Radiation Medicine, State Key Laboratory of Natural and Biomimetic Drugs, School of Basic Medical Sciences, Peking University, Beijing 100191, China; 3 Key Laboratory of Protein and Peptide Pharmaceuticals, CAS Center for Excellence in Biomacromolecules, Institute of Biophysics, Chinese Academy of Sciences, Beijing 100101, China; 4 Beijing Translational Center for Biopharmaceuticals, Beijing 100101, China

**Keywords:** Nanobody, Sortase A, Site-specific radiolabeling, ^99m^Tc

## Abstract

Recently, there has been a lot of interest by using nanobodies (heavy chain-only antibodies produced naturally from the *Camelidae*) as targeting molecules for molecular imaging, especially for the nuclear medicine imaging. A radiolabeled method that generates a homogeneous product is of utmost importance in radiotracer development for the nuclear medicine imaging. The conventional method for the radiolabeling of nanobodies is non-specifically, which conjugates the radioisotope chelating group to the side chain ɛ-amine group of lysine or sulfhydryl of cysteine of nanobodies, with a shortcoming of produce of the heterogeneous radiotracer. Here we describe a method for the site-specific radioisotope ^99m^Tc labeling of nanobodies by transpeptidase Sortase A. The radiolabeling process includes two steps: first step, NH_2_-GGGGK(HYNIC)-COOH peptide (GGGGK = NH_2_-Gly-Gly-Gly-Gly-Lys-COOH, HYNIC = 6-hydrazinonicotinyl) was labeled with ^99m^Tc to obtain GGGGK-HYNIC-^99m^Tc; second step, Sortase A catalyzes the formation of a new peptide bond between the peptide motif LPETG (NH_2_-Leu-Pro-Glu-Thr-Gly-COOH) expressed C-terminally on the nanobody and the N-terminal of GGGGK-HYNIC-^99m^Tc. After a simple purification process, homogeneous single-conjugated and stable ^99m^Tc-labeled nanobodies were obtained in >50% yield. This approach demonstrates that the Sortase A-mediated conjugation is a valuable strategy for the development of site-specifically ^99m^Tc-labeled nanobodies.

## INTRODUCTION

**Figure 1:**
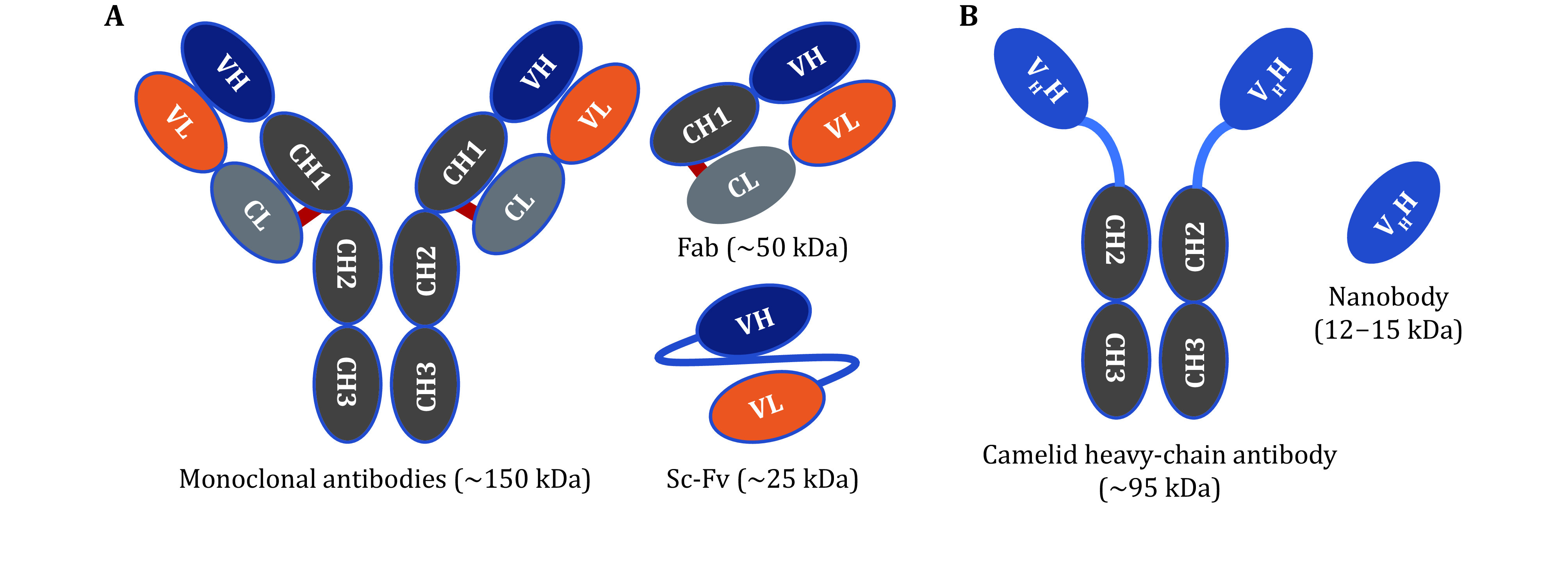
The schematic representation of different types of antibodies. **A** The structure of antibody and its fragments, Fab and sc-Fv. **B** Camelid heavy-chain antibody and its variable region, nanobody. Adapted from Liu *et al*. (2021)

Recently, a new class of variable region of the heavy-chain-only antibodies (V_H_H) derived from *Camelidae*, referred to as nanobody (Nb) (Hamers-Casterman *et al*. 1993), has gained a growing interest in the field of molecular imaging, given their peculiar features and high versatility (Yang and Shah 2020). The main advantages of Nb as molecular probes are as follows: (1) Compared with the monoclonal antibodies (mAbs, ~150 kDa), antigen fragment (Fab, ~50 kDa) and single chain Fv (sc-Fv, ~25 kDa), Nb has the smallest molecular weight (12–15 kDa) (Fig. 1) (Oliveira *et al*. 2013). Due to their small size and the absence of Fc fragment, Nb is rapidly eliminated from the circulation, and can be cleared quickly through the kidney, which results in a significantly reduced background and increased signal-to-noise ratio as early as 1 h after tracer injection (Gao *et al*. 2020); (2) Compared with peptides, Nb has high affinity and specificity. The antigen binding affinity of Nb is more than 10–100 times to peptides, which is close to mAbs (Hassanzadeh-Ghassabeh *et al*. 2013); (3) The immunogenicity and toxicity of Nb are very low, and they are not as prone to adhesion as sc-Fv (Keyaerts *et al*. 2016); (4) Nb has good tissue penetration and can be fully combined to targeted tissues; (5) By using modern genetically engineered antibody technology, high yield Nb can be obtained (McMahon *et al*. 2018), whose structure can also be easily modified, making it an ideal targeting molecule candidate for nuclear medicine imaging agents.

The previously reported methods of Nb radiolabeling are usually accomplished by using the side chain primary amine of lysine residues or sulfhydryl of cysteine residues of Nb (Lv *et al*. 2020), but these methods have some limitations. Nb usually has multiple solvent-exposed lysine, making it difficult to control where and how many radioisotopes are labeled. In addition, the presence of lysine residues at or near the target antigen-binding site can lead to reduced Nb activity after conjugation (Alt *et al*. 2014). In order to avoid the heterogeneity of the tracer, some studies have introduced the unpaired cysteine at the C-terminal of Nb for site-specific labeling (Feng *et al*. 2020). However, this strategy requires the reducing agent to liberate the introduced cysteine residue. These reducing agents must be carefully titrated to prevent the breakdown of disulfide bonds within Nb, which are essential for stability and may lead to unnecessary reduction by-products. Other methods under investigation for designing site-specific labeling of Nb are alkyne-azide click reactions, which involve the insertion of unnatural amino acids into the nanobody structure (Agarwal and Bertozzi 2015). In addition, ^99m^Tc-tricarbonyl reacts site-specifically with a genetically inserted C-terminal hexahistidine tag (His_6_) of nanobody for ^99m^Tc labeling (Xing *et al*. 2019). However, ^99m^Tc-tricarbonyl is unstable and easy decomposition (Biechlin *et al*. 2005).

**Figure 2:**
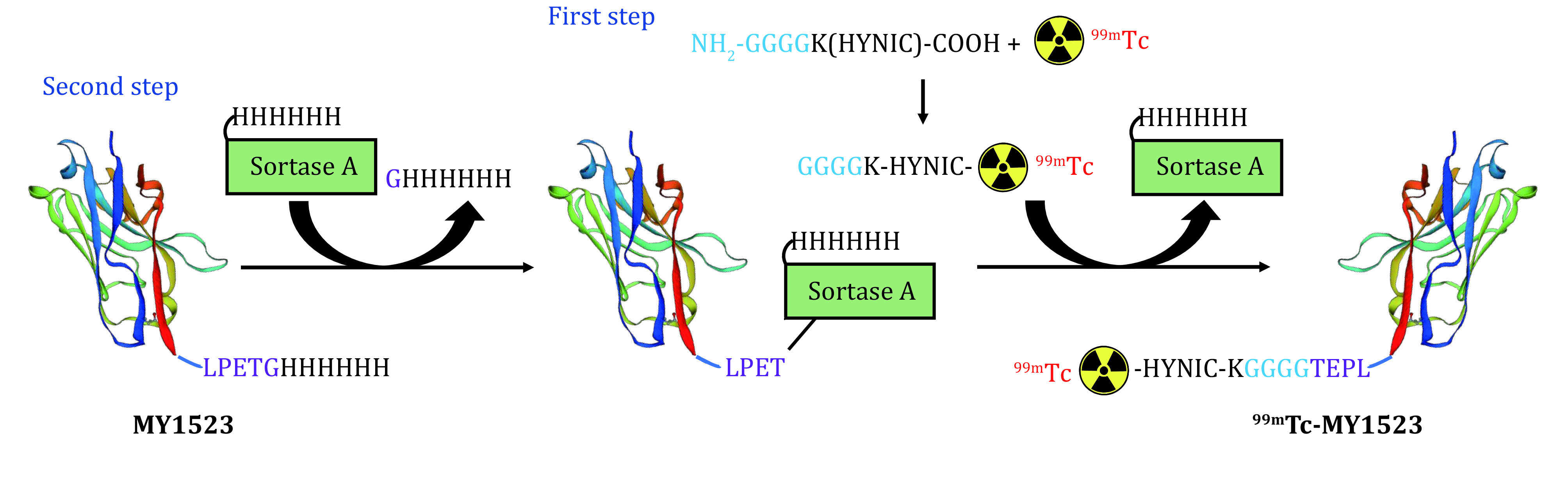
The schematic diagram of ^99m^Tc labeled MY1523 by Sortase A

Sortase A (SrtA), a transpeptidase, is derived from *Staphylococcus aureus* that has been extensively used for protein engineering and antibody modification (Paterson *et al*. 2014; Popp *et al*. 2007). SrtA recognizes substrate proteins bearing a short motif (LPXTG) of C-terminal and cleaves the peptide between threonine and glycine forming a new bond with the nucleophiles containing N-terminal oligo-glycine motif (Mazmanian *et al*. 1999). Several studies have reported the use of SrtA for the site-specific labeling of Nb (Massa *et al*. 2016; Rashidian *et al*. 2016). For example, Massa *et al*. demonstrated SrtA-mediated the site-specific indium-111 and gallium-68 labeling of human epidermal growth factor receptor 2 (HER2)-targeting nanobody (Massa *et al*. 2016). Since nearly 85% of diagnostic radiotracers currently available in clinical nuclear medicine are ^99m^Tc-compounds due to the ideal nuclear properties of ^99m^Tc, as well as their widespread availability using commercially available ^99m^Tc-generators (Pietzsch *et al*. 2013). Here, we describe a generic method for SrtA-mediated site-specific ^99m^Tc labeling of Nb, while using the programmed death ligand-1 (PD-L1)-targeting nanobody (MY1523). First step, NH_2_-GGGGK(HYNIC)-COOH peptide was labeled with ^99m^Tc using TPPTS and tricine as co-ligands to obtain trinary ^99m^Tc-radiolabed complex of (^99m^Tc-(HYNIC-peptide) (TPPTS)(tricine)) (termed as GGGGK-HYNIC-^99m^Tc). This trinary ^99m^Tc-radiolabed complex have been reported to have good stability (Jia *et al*. 2006). Second step, the SrtA catalyzes the formation of a new peptide bond between the peptide motif LPETG expressed C-terminally on the MY1523 and the N-terminal of GGGGK-HYNIC-^99m^Tc (Fig. 2). This enzyme-mediated ligation is a more elegant method which avoids Nb to contact violent labeling conditions. We expect this labeling protocol to be resulted in a homogeneous, site-specifically single-conjugated, and stable ^99m^Tc-labeled nanobody.

## SUMMARIZED PROCEDURE

( 1 ) Synthesize NH_2_-GGGGK(HYNIC)-COOH;

( 2 ) Prepare ^99m^Tc-labeling kit;

( 3 ) Prepare GGGGK-HYNIC-^99m^Tc;

( 4 ) Determine the radiochemical yield (RCY) of GGGGK-HYNIC-^99m^Tc by high performance liquid chromatography (HPLC);

( 5 ) Prepare the ^99m^Tc-MY1523 by labeling MY1523-LPETG-His_6_ with GGGGK-HYNIC-^99m^Tc;

( 6 ) Determine the RCY of ^99m^Tc-MY1523 by instant thin-layer chromatography (ITLC);

( 7 ) Purify the ^99m^Tc-MY1523 by high-performance size exclusion chromatography (HPSEC);

( 8 ) Determine the radiochemical purity (RCP) of ^99m^Tc-MY1523 by ITLC;

( 9 ) Assess the *in vitro* stability of ^99m^Tc-MY1523;

(10) Assess the *in vivo* stability of ^99m^Tc-MY1523.

## PROCEDURE

### Synthesis of NH_2_-GGGGK(HYNIC)-COOH [TIMING 3-4 d]

( 1 ) The semi-preparative HPLC method (Method 1) used the Agilent 1260 HPLC system equipped with a UV/vis detector (*λ* = 210 or 254 nm) and C_18_ column (250 × 10 mm I.D. S-5 μm, 12 nm). The flow rate was 2.5 mL/min with a gradient mobile phase going from 80% A (0.05% TFA in water) and 20% B (0.05% TFA in acetonitrile) at 0 min to 40% B at 20 min, and 20% B at 22 min.

( 2 ) Dissolve Fmoc-GGGGK-COOH (10.0 mg, 16.8 μmol), HYNIC-NHS (10.5 mg, 25.1 μmol) in N,N-dimethylformamide (DMF, 500 μL) and mixed with N,N-diisopropyletylamine (DIPEA, 50 μmol).

( 3 ) Incubate the mixture for 6 h at room temperature (RT).

**[CRITICAL STEP]** It is important to allow the reaction to last for 6 h, so that the reaction is fully completed.

( 4 ) Terminate the reaction with NH_4_OAc buffer (1 mL, 100 mmol/L, pH = 7.0).

( 5 ) Purify Fmoc-GGGGK(HYNIC)-COOH by semi-preparative HPLC (Method 1), and collect the fractions at 17.2 min on the HPLC.

( 6 ) The desired product collected from the fractions was identified by MALDI-TOF-MS, and the Fmoc-GGGGK (HYNIC)-COOH was lyophilized and stored.

**[PAUSE POINT]** Store the lyophilized Fmoc-GGGGK(HYNIC)-COOH at −20 °C until it is needed.

( 7 ) Dissolve Fmoc-GGGGK(HYNIC)-COOH (9.0 mg, 10 μmol) in 400 μL 20% Piperidine-DMF.

( 8 ) Incubate the mixture for 25 min at RT.

**[CRITICAL STEP]** It is highly recommended to maintain the duration of the reaction between 20–30 min. The extension of the reaction time may produce by-products.

( 9 ) The reaction was terminated with a NH_4_OAc buffer (1 mL, 100 mmol/L, pH = 7.0).

(10) Purify NH_2_-GGGGK(HYNIC)-COOH by semi-preparative HPLC (Method 1) and collect the fractions at 10.5 min on the HPLC.

(11) The desired product collected from the fractions was identified by MALDI-TOF-MS, and the NH_2_-GGGGK(HYNIC)-COOH was lyophilized and stored.

**[PAUSE POINT]** The lyophilized product can be stored at −20 °C as powder indefinitely.

### Preparation of ^99m^Tc-labeling kit [TIMING 1-2 d]

(12) Dissolve tricine (6.5 mg), trisodium triphenylphosphine-3,3’,3’’-trisulfonate (TPPTS, 5 mg), NH_2_-GGGGK(HYNIC)-COOH (10 μg), succinic acid (12.7 mg), and disodium succinate (38.5 mg) in water (1 mL), and add to a 10 mL glass bottle.

(13) Lyophilize the mixture to get the labeling kit.

**[PAUSE POINT]** The lyophilized kit can be stored at −20 °C as indefinitely.

### Preparation of GGGGK-HYNIC-^99m^Tc [TIMING 0.5 h]

(14) Add Na^99m^TcO_4_ solution (1 mL, 370–740 MBq) to a labeling kit.

(15) The labeling kit was incubated in water bath at 100 °C for 25 min.

**[CRITICAL STEP]** It is highly recommended to maintain the heating time between 20–30 min.

(16) Cool the labeling kit to RT.

**[CAUTION!]** It is imperative to obtain appropriate training from the institutional radiation safety office before experimenting with radioactivity. When handling radioactive materials, please comply with all relevant regulations and use appropriate protective measures.

### Determination of the radiochemical yield of GGGGK-HYNIC-^99m^Tc [TIMING 0.5 h]

(17) The radio-HPLC method (Method 2) (Luo *et al*. 2020) using the Agilent 1260 HPLC system was equipped with a radioactive detector and C_18_ column (250 × 4.6 mm I.D. S-5 μm, 12 nm). The flow rate was 1.0 mL/min with a gradient mobile phase going from 90% solution A (0.05% TFA in water) and 10% solution B (0.05% TFA in acetonitrile) at 0 min to 40% solution B at 17.5 min, and to 10% solution B at 20 min.

(18) Pre-equilibrate a C_18_ column with 20 mL of 90% solution A and 10% solution B, at a rate of 1.0 mL/min.

(19) Use the radio-HPLC (Method 2) to determine the radiochemical yield (RCY) of GGGGK-HYNIC-^99m^Tc.

(20) The RCY was calculated by expressing the peak corresponding to GGGGK-HYNIC-^99m^Tc as a percentage of the total activity in the radio-HPLC chromatogram.

### Preparation of ^99m^Tc-MY1523 [TIMING 0.75 h]

(21) Adjust the pH of GGGGK-HYNIC-^99m^Tc solution to 7–8 with 2 mol/L NaOH water solution (50 μL).

**[CRITICAL STEP]** Since the optimal pH for SrtA enzymatic catalysis is 7–8, it is necessary to adjust the pH of GGGGK-HYNIC-^99m^Tc solution.

(22) Mix GGGGK-HYNIC-^99m^Tc solution (185 MBq), MY1523-LPETG-His_6_ (100 μg, 2 mg/mL), SrtA (50 μg, 2 mg/mL), and 1 mol/L CaCl_2_ water solution (10 μL).

**[CRITICAL STEP]** The molar ratio between GGGGK-HYNIC-^99m^Tc and MY1523-LPETG-His_6_ is highly recommended to be 1∶5.

**[? TROUBLESHOOTING]**

(23) Incubate the mixture for 25 min at RT.

**[CRITICAL STEP]** It is highly recommended to maintain the duration of the reaction between 25–30 min. Prolonging the reaction time will reduce the labeling efficiency.

### Determination of the radiochemical yield of ^99m^Tc-MY1523 [TIMING 0.2 h]

(24) Check the RCY of ^99m^Tc-MY1523 by instant thin-layer chromatography (ITLC). ITLC was performed on silica gel (ITLC-SG, 1.5 cm × 10 cm strips) using saline as the developing solution.

(25) Drop a sample of approximately 0.37 MBq onto the starting line of ITLC-SG strip (1.5 cm from the bottom line) and let it dry.

(26) Allow saline to migrate to the front edge of the ITLC-SG strip (1 cm from the top), then take out the strip and let it dry.

(27) Use a radio-TLC scanner (Bioscan AR2000) to scan the ITLC-SG paper.

(28) Analyze the ITLC chromatograms: Rf = 0–0.3 for ^99m^Tc-MY1523, Rf = 0.7–1.0 for GGGGK-HYNIC-^99m^Tc and free ^99m^Tc.

(29) The RCY was calculated by expressing the peak corresponding to the Rf of ^99m^Tc-MY1523 as percentage of the total activity on the ITLC chromatograms.

**[? TROUBLESHOOTING]**

### Purification of the ^99m^Tc-MY1523 [TIMING 1 h]

(30) The high-performance size exclusion chromatography (HPSEC) method (Method 3) used the Agilent 1260 HPLC system equipped with a UV/vis detector (*λ* = 280 nm), radioactive detector and Superdex-75^TM^ column (Increase 10/300 GL). The flow rate was 0.8 mL/min with a gradient mobile phase going from 100% A (PBS (pH = 7.2−7.4)) at 0 min to 100% A at 50 min.

(31) Pre-equilibrate a Superdex-75^TM^ column with 50 mL of PBS (pH = 7.2–7.4), at a rate of 0.8 mL/min.

(32) Purify the ^99m^Tc-MY1523 by HPSEC (Method 3). Inject the labeled solution and maintain a flow rate of 0.8 mL/min. Collect the fractions at 15–18 min.

[**CRITICAL STEP**] It is highly recommended to collect the fractions at 200 μL/tube.

**[? TROUBLESHOOTING]**

### Determination of the radiochemical purity of ^99m^Tc-MY1523 [TIMING 1 h]

(33) Check the RCP of ^99m^Tc-MY1523 by ITLC. ITLC was performed on ITLC-SG (1.5 cm × 10 cm strips) using saline as the developing solution.

(34) Drop a sample of approximately 0.37 MBq onto the starting line of ITLC-SG strip (1.5 cm from the bottom line) and let it dry.

(35) Allow saline to move to the front edge of the ITLC-SG strip (1 cm from the top), then take out the strip and let it dry.

(36) Use a radio-TLC scanner (Bioscan AR2000) to detect the ITLC-SG strip.

(37) Analyze the ITLC chromatograms: Rf = 0–0.3 for ^99m^Tc-MY1523, Rf = 0.7–1.0 for GGGGK-HYNIC-^99m^Tc and free ^99m^Tc.

(38) The RCP was calculated by expressing the peak corresponding to the Rf of ^99m^Tc-MY1523 as percentage of the total activity on the ITLC chromatograms.

(39) The RCP of ^99m^Tc-MY1523 was also determined by HPSEC (Method 3).

(40) The RCY was calculated by expressing the peak corresponding to ^99m^Tc-MY1523 as percentage of the total activity in the HPSEC chromatogram.

### Assessment of the *in vitro* stability of ^99m^Tc-MY1523 [TIMING 24 h]

(41) Mix purified ^99m^Tc-MY1523 solution (about 9 MBq, 100 μL) with mouse serum (900 μL).

(42) Incubate the mixture for 0, 1, 2, 4, 8 and 24 h at RT.

(43) Determine the RCP of ^99m^Tc-MY1523 as described in Steps (33)–(38).

(44) Assess the *in vitro* stability of ^99m^Tc-MY1523 according to the RCP of radiotracer.

### Assessment of the *in vivo* stability of ^99m^Tc-MY1523 [TIMING 6-7 h]

(45) Inject purified ^99m^Tc-MY1523 solution (about 18 MBq, 200 μL) into ICR mice via the tail vein.

(46) Collect the urine of mice at 6 h post-injection (p.i.).

(47) Determine the RCP of ^99m^Tc-MY1523 as described in Steps (33)–(38).

(48) Assess the *in vivo* stability of ^99m^Tc-MY1523 according to the RCP of radiotracer.

**[TIMING]**

Step 1–11 Synthesis of NH_2_-GGGGK(HYNIC)-COOH takes about 3–4 d.

Step 12–13 Preparation of ^99m^Tc labeling kit takes approximately 1–2 d.

Step 14–20 Preparation and determination of the radiochemical yield of GGGGK-HYNIC-^99m^Tc take approximately 1 h.

Step 21–29 Preparation and determination of the radiochemical yield of ^99m^Tc-MY1523 take approximately 1 h.

Step 30–32 Purification of the ^99m^Tc-MY1523 takes approximately 1 h.

Step 33–40 Determination of the radiochemical purity of ^99m^Tc-MY1523 takes approximately 1 h.

Step 41–44 Assessment of the *in vitro* stability of ^99m^Tc-MY1523 takes approximately 24 h.

Step 45–48 Assessment of the *in vivo* stability of ^99m^Tc-MY1523 takes approximately 6–7 h.

**[? TROUBLESHOOTING]**

Step 22 When CaCl_2_ water solution is added, white fluffy precipitate was detected in the mixed solution. This is Ca^2+^ precipitate (Ga_3_(PO_4_)_2_). We can centrifuge the mixture, take the supernatant and continue the labeling reaction.

Step 29 If the labeling efficiency is very low, it may be that, (1) insufficient GGGGK-HYNIC-^99m^Tc is added to the reaction, (2) pH of the reaction solution is not compatible with SrtA activity, or (3) SrtA is inactive. We can try that, (1) increase the amount of GGGGK-HYNIC-^99m^Tc, (2) ensure the pH of the reaction solution is 7–8, or (3) use new SrtA.

Step 32 Less purified ^99m^Tc-Nanobody were collected. First, determine whether the radiochemical yield was within the expected range (>50%). One possibility is that the nanobody was stuck on the Superdex-75^TM^ column. In this case, using 0.1% Tween-20–PBS (pH = 7.2–7.4) as mobile phase can help flush out the radiolabeled nanobody.

## ANTICIPATED RESULTS

**Figure 3:**
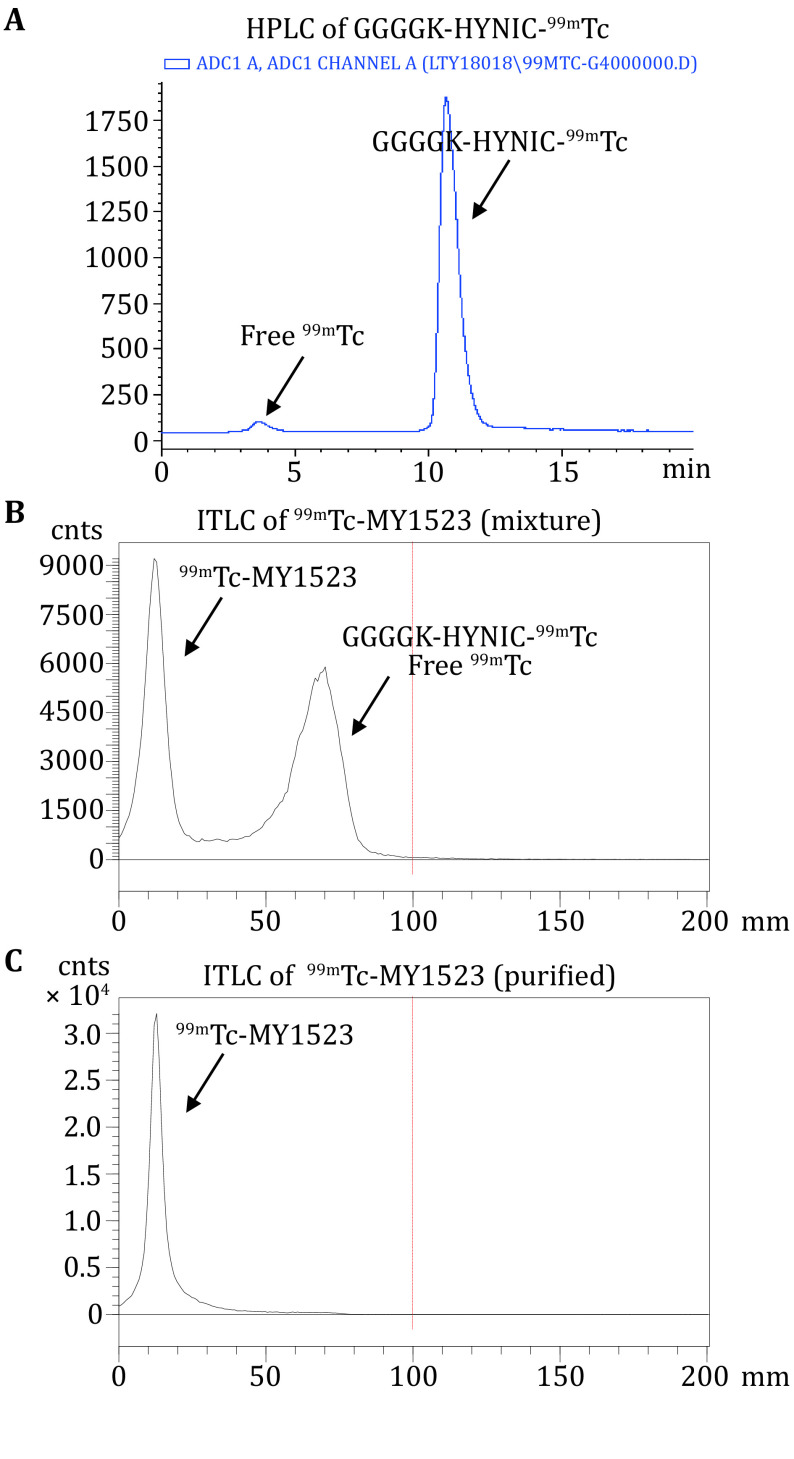
The radiochemistry of GGGGK-HYNIC-^99m^Tc and ^99m^Tc-MY1523. **A** Representative RP-HPLC chromatogram of GGGGK-HYNIC-^99m^Tc. **B** Representative ITLC chromatograms of the ^99m^Tc-MY1523 mixture. **C** The purified product of ^99m^Tc-MY1523

**Figure 4:**
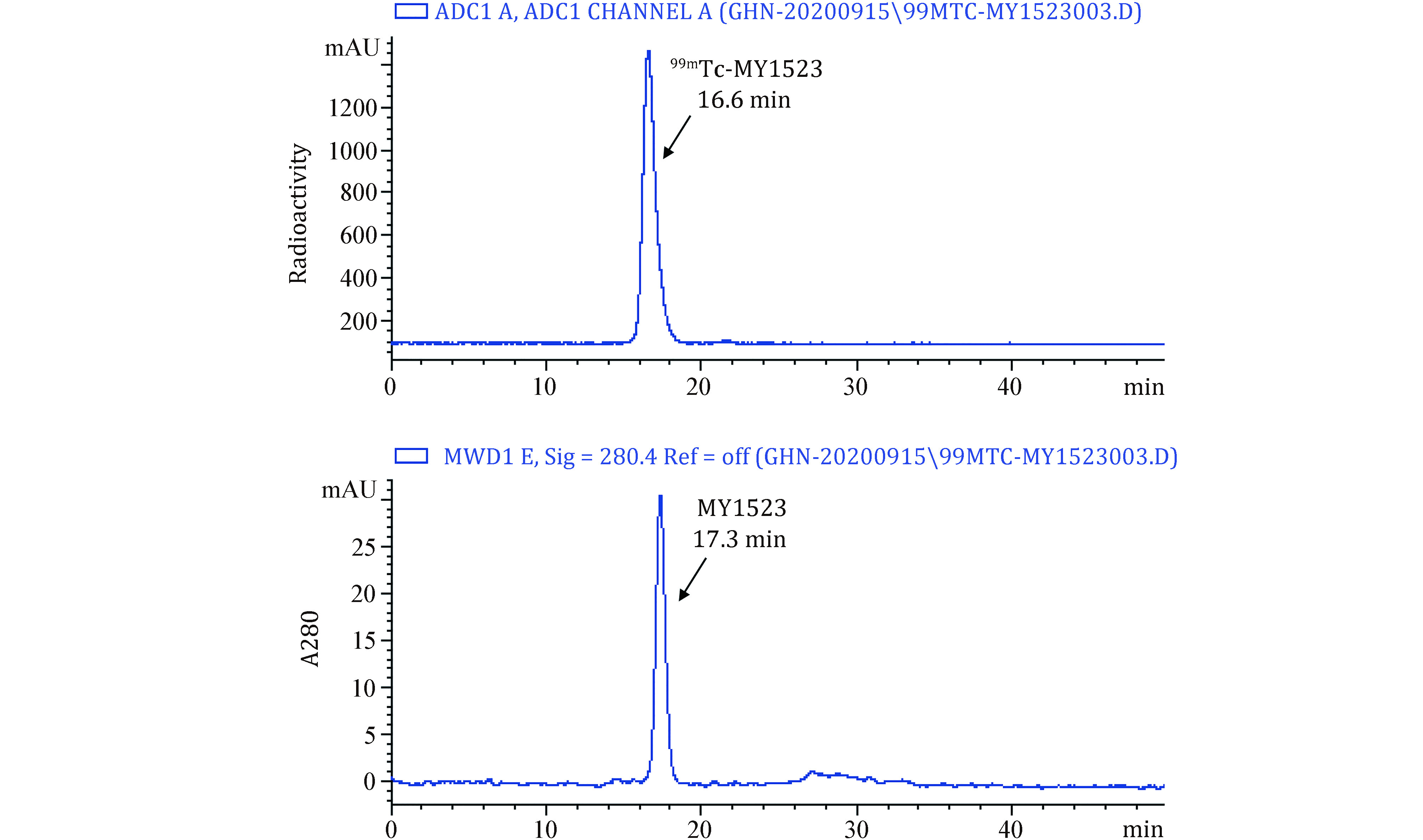
The representative HPSEC chromatogram of ^99m^Tc-MY1523

Figures 3 and 4 present typical representative data obtained using the method described here. SrtA mediated the site-specific radionuclide ^99m^Tc labeling of nanobody.

**Figure 5:**
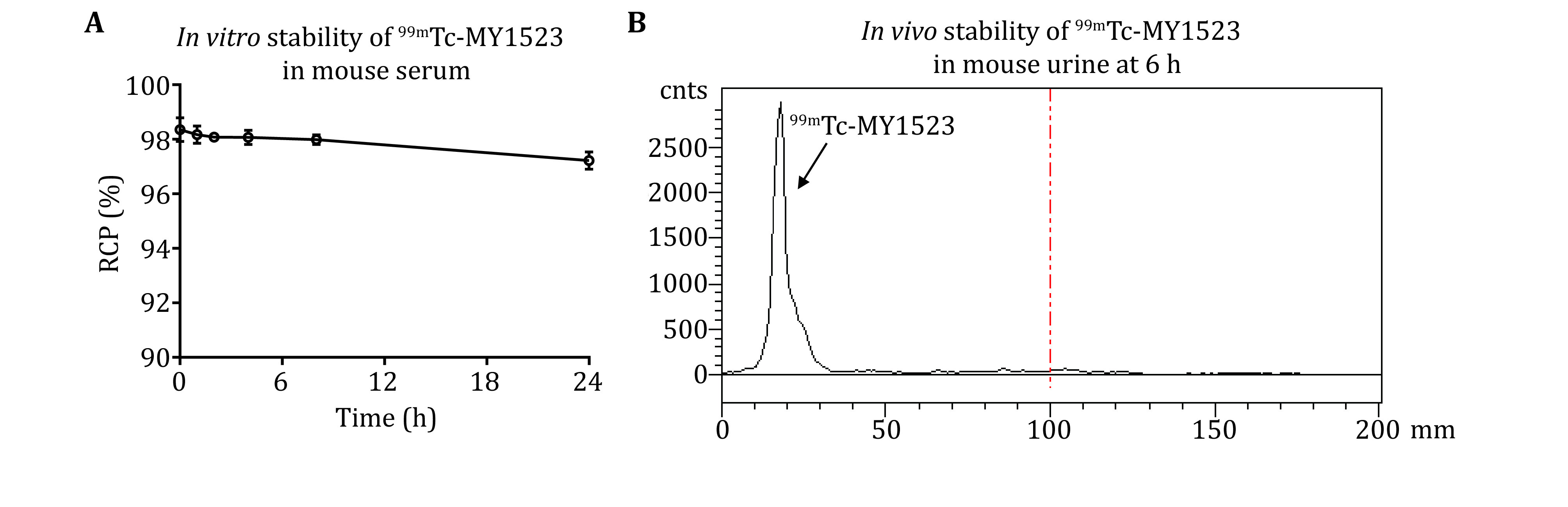
The stability of ^99m^Tc-MY1523. **A** *In vitro* stability of ^99m^Tc-MY1523 in mouse serum. **B** *In vivo* stability of ^99m^Tc-MY1523 in mouse urine at 6 h p.i.

The radiochemical yield (RCY) of GGGGK-HYNIC-^99m^Tc was determined by RP-HPLC. The representative HPLC chromatogram of GGGGK-HYNIC-^99m^Tc was shown in Fig. 3A. The RCY of GGGGK-HYNIC-^99m^Tc was >95%. The RCY of ^99m^Tc-MY1523 was determined by ITLC. The representative ITLC chromatogram was shown in Fig. 3B. As results, 50% RCY was generally obtained after the two steps in total. After purification, the radiochemical purity (RCP) of the final product was determined by ITLC. The representative ITLC chromatogram of ^99m^Tc-MY1523 was shown in Fig. 3C. The RCP of end-product was >95%. The specific activity of ^99m^Tc-MY1523 was >11.0 MBq/nmol. The RCP of ^99m^Tc-MY1523 was also determined by HPSEC. Fig. 4 shows a typical representative HPSEC chromatogram. The RCP of ^99m^Tc-MY1523 was >95%. The retention time of ^99m^Tc-MY1523 was at 16.6 min, which was a little earlier than that of cold MY1523 (17.3 min for MY1523). As shown in Fig. 5A, ^99m^Tc-MY1523 was stable in mouse serum at room temperature for 24 h. The RCP of urine sample collected at 6 h p.i. was >95% (Fig. 5B), indicating that ^99m^Tc-MY1523 has good *in vivo* stability.

## MATERIALS AND EQUIPENT

### Reagents

• Fmoc-GGGGK-COOH (Shanghai GL Biochem Ltd.)

• HYNIC-NHS (Shanghai GL Biochem Ltd.)

• N,N-dimethylformamide (DMF; Sigma-Aldrich, cat. no. 1003972500)

**[CAUTION!]** DMF is flammable and toxic.

• N,N-diisopropyletylamine (DIPEA; Sigma-Aldrich, cat. no. 8008940250)

**[CAUTION!]** DIPEA is highly flammable and corrosive.

• Acetonitrile (Honeywell, cat. no. AH015-4)

**[CAUTION!]** Acetonitrile is flammable and toxic.

• Trifluoroacetic acid (TFA; Sigma-Aldrich, cat. no. 302031)

**[CAUTION!]** TFA is strongly corrosive and toxic.

• N-Tris[Hydroxymethyl]Methylglycine (Tricine, Sigma-Aldrich, cat. no. T0377)

• Trisodium triphenylphosphine-3,3’,3’’-trisulfonate (TPPTS; J&K Scientific, cat. no. T895938)

• Succinic acid (Sigma-Aldrich, cat. no. 14080)

• Disodium succinate (Sigma-Aldrich, cat. no. 224731)

• Na^99m^TcO_4_ (Beijing Atomic High-Tech Co., Ltd., China)

• Sortase A (Shanghai Novamab Biopharmaceuticals Co, Ltd, China)

• MY1523-LPETG-His_6_ (Shanghai Novamab Biopharmaceuticals Co, Ltd, China)

• Tween-20 (Sigma-Aldrich, cat. no. P9416)

• Mouse serum (Shanghai Yeasen Biotechnology Co., Ltd, cat. no. 36118ES08)

• Piperidine (Sigma-Aldrich, cat. no. 80645)

• Saline (Shijiazhuang No.4 Pharmaceutical Co, Ltd)

• Ammonium acetate (NH_4_OAc, Sigma-Aldrich, cat. no. 32301)

• Ammonium hydroxide (Sigma-Aldrich, cat. no. 221228)

• Sodium hydroxide (NaOH, Sigma-Aldrich, cat. no. 221465)

• Calcium chloride (CaCl_2_, Sigma-Aldrich, cat. no. 499609)

• Phosphate buffer saline (PBS, Biological Industries, cat. no. 02-024-1ACS)

### Animals

• Female ICR mice (5 weeks of age, Beijing Vital River Laboratory Animal Technology Co., Ltd.)

### Equipment

• HPLC system (Agilent 1260 series)

• HPLC radioactive detector (Elysia-Raytest, Germany)

• Semi-preparative C_18_ column (250 × 10 mm I.D. S-5 μm, 12 nm, YMC-Pack ODS-A, cat. no. 110EA70231)

• Analytical C_18_ column (250 × 4.6 mm I.D. S-5 μm, 12 nm, YMC-Pack ODS-A, cat. no. 121GA70148)

• Radio-TLC imaging scanner (Bioscan, USA, cat. no. AR-2000)

• Radioactivity meter (Capintec Inc., USA, cat. no. CRC-15R)

• Superdex-75^TM^ size exclusion chromatography column (Increase 10/300 GL, GE Healthcare)

• Electric-heated thermostatic water bath (Shanghai Senxin Experimental Instrument Co, Ltd, cat. no. DK-S12)

• Dry bath incubator (Fisher Scientific, cat. no. 11-718-2)

• ITLC-SG chromatograpy paper (10 cm long and 1.5 cm wide, Agilent Technologies, cat. no. SGI0001)

• pH paper (Aladdin Inc.)

• Lyophilizer (Beijing Boyikang Experimental Instrument Co., Ltd, cat. no. FD-1D-50)

• Glass bottle, 10 mL (Agilent Technologies, cat. no. 5190-2241)

• Centrifuge tubes, 1.5 mL (Corning Life Sciences Co. Ltd, cat. no. MCT-150-C)

• Matrix-assisted laser desorption/ionization time of flight mass spectrometry (MALDI-TOF-MS, Bruker, Germany)

### Reagent setup

• 0.05% TFA–water (*v*/*v*). Mix 2 mL of TFA with 4 L of water. Filter the solution and store it at 4 °C for up to three months.

• 0.05% TFA–acetonitrile (*v*/*v*). Mix 2 mL of TFA with 4 L of acetonitrile. Filter the solution and store it at RT (21–25 °C) for up to three months.

• 100 mmol/L NH_4_OAc buffer (pH = 7.0). Use ammonium hydroxide to adjust the pH of 100 mmol/L NH_4_OAc water solution to 7. Filter the buffet and store it at 4 °C for up to three months.

• 20% Piperidine–DMF (*v*/*v*). Mix 20 mL of piperidine with 80 mL of DMF. The Fmoc deprotection solution can be stored at RT for one month.

• 0.1% Tween-20–PBS (*v*/*v*). Mix 1 mL of Tween-20 with 1 L of PBS. Filter the solution and store it at 4 °C for up to three months.

## Conflict of interest

Qi Luo, Hannan Gao, Jiyun Shi and Fan Wang declare that they have no conflict of interest.
